# Aerogels Based on Reduced Graphene Oxide/Cellulose Composites: Preparation and Vapour Sensing Abilities

**DOI:** 10.3390/nano10091729

**Published:** 2020-08-31

**Authors:** Yian Chen, Petra Pötschke, Jürgen Pionteck, Brigitte Voit, Haisong Qi

**Affiliations:** 1 Leibniz-Institut für Polymerforschung Dresden e. V. (IPF), 01069 Dresden, Germany; chen@ipfdd.de (Y.C.); pionteck@ipfdd.de (J.P.); voit@ipfdd.de (B.V.); 2 Organic Chemistry of Polymers, Faculty of Chemistry and Food Chemistry, Technische Universität Dresden, 01062 Dresden, Germany; 3 State Key Laboratory of Pulp and Paper Engineering, South China University of Technology, Guangzhou 510640, China; 4 Guangdong Engineering Research Center for Green Fine Chemicals, South China University of Technology, Guangzhou 510640, China

**Keywords:** cellulose, reduced graphene oxide, conductive polymer composite, aerogel, vapour sensor

## Abstract

This paper reports on the preparation of cellulose/reduced graphene oxide (rGO) aerogels for use as chemical vapour sensors. Cellulose/rGO composite aerogels were prepared by dissolving cellulose and dispersing graphene oxide (GO) in aqueous NaOH/urea solution, followed by an in-situ reduction of GO to reduced GO (rGO) and lyophilisation. The vapour sensing properties of cellulose/rGO composite aerogels were investigated by measuring the change in electrical resistance during cyclic exposure to vapours with varying solubility parameters, namely water, methanol, ethanol, acetone, toluene, tetrahydrofuran (THF), and chloroform. The increase in resistance of aerogels on exposure to vapours is in the range of 7 to 40% with methanol giving the highest response. The sensing signal increases almost linearly with the vapour concentration, as tested for methanol. The resistance changes are caused by the destruction of the conductive filler network due to a combination of swelling of the cellulose matrix and adsorption of vapour molecules on the filler surfaces. This combined mechanism leads to an increased sensing response with increasing conductive filler content. Overall, fast reaction, good reproducibility, high sensitivity, and good differentiation ability between different vapours characterize the detection behaviour of the aerogels.

## 1. Introduction

Volatile organic compounds (VOCs) are organic chemicals with high vapour pressure at room temperature. Some VOCs are dangerous to human health or cause harm to the environment. Therefore, reliable, portable, and low-cost vapour sensors or gas sensors play an increasingly important role in numerous applications in industrial production and safety, air quality monitoring, medical diagnosis, military, space exploration, etc. [1,2,3,4,5,6,7]. In the development of vapour/gas sensing materials, the focus today is on promoting the combination of properties, such as ultrahigh sensitivity, fast response and recovery, high specificity, and good reversibility [8,9]. Thus far, numerous sensing materials have been reported, for example, semiconducting metal oxide (SMO) [10], nano-carbons, e.g., carbon nanotubes and graphene [11], organic semiconductors [12], intrinsically conducting polymers (ICPs) [13,14,15], and conductive polymer composites (CPCs) [16].

The most important characteristic that determines the sensitivity of nanometric materials is their high surface-to-volume ratio. This is beneficial for the adsorption of vapours/gases on the sensing material and it can enhance the sensitivity of the sensing material. Graphene, a single sheet of sp^2^-hybridized carbon atoms, has attracted tremendous interest because of its outstanding mechanical properties [17], chemical stability [18], superior electrical conductivity [19], and strong vapour/gas adsorption capacity [20]. Thus, this interesting material shows great potential for many applications, such as supercapacitors [21], lithium ion batteries [22], solar cells [23], and vapour or gas sensors [24]. The two-dimensional structure of graphene makes the electron transport through graphene highly sensitive to the adsorption of vapour/gas molecules [25], which is restricted upon such adsorption. All of these features of graphene are beneficial for its sensing properties, making it an ideal candidate for vapour/gas detecting. In addition, in conductive polymer composites (CPCs) made of isolating polymers with conductive graphene, the swelling of the polymer matrix that is caused by vapour or solvents destroys the conductive pathways in the CPC by disconnection of the filler-filler contacts and increasing the filler-filler distances above the tunnelling distance. This hampers the electron transport between the graphene sheets leading to a decrease in the electrical conductivity with increasing matrix swelling degree, which is the primary vapour sensing mechanism in CPCs.

The practical application of graphene is severely restricted by its poor dispersion and secondary agglomeration during processing, which makes it difficult to maintain its superiority. There have been many efforts to deal with the problem of obtaining high surface area graphene and hindering its re-agglomeration, including chemical vapour deposition, micromechanical exfoliation of graphite, the reduction of graphene oxide (GO), etc. [26,27,28,29]. Among them, the reduction of GO is the most promising method for the cost-effective, large-scale production of graphene-based materials. The presence of oxygen-containing groups in GO makes it highly hydrophilic and water soluble and allows for the chemical modification of graphene.

It is known that the vapour or gas sensing performance is greatly enhanced by increasing the internal surface area between vapour molecules and the vapour sensor material [30]. Aerogel is a synthetic ultralight material that has a porous solid network with air pockets [31,32]. Due to their unique features such as large open pores, low density, and high internal surface area, aerogels are very suitable for vapour adsorption and storage. Moreover, different types of aerogels are also suitable for other applications, such as photocatalyst [33], strain and pressure sensor [34], and vapour sensor [35,36]. In our previous work, cellulose/rGO composite hydrogels were successfully prepared by dissolving cellulose and dispersing GO in NaOH/urea aqueous solution, followed by the in-situ reduction of GO to rGO using vitamin C as reducing agent [37]. The composite aerogels were obtained by the freeze-drying method. Overall, the preparation of these sensory aerogels with highly porous networks is a simple, efficient, and “green” process. It could be exemplarily shown that such aerogels are suitable for a variety of sensing applications, such as temperature and humidity sensing, strain sensing, and sensing of liquids.

Herein, cellulose/rGO composite aerogels with different rGO contents were fabricated by the freeze-drying of cellulose/rGO composite hydrogels. The sensing properties of the resultant aerogels were determined by their multiple exposures to vapour/dry air cycles. The influences of overall rGO content, vapour type, and vapour concentration on the sensing response have been analysed.

## 2. Experimental

### 2.1. Materials

Cotton linters (DP 500) were used as the cellulose material, which was bought from Hubei Chemical Fiber Group Ltd. (Xiangfan, China). GO dispersion (4 mg/mL, monolayer content > 95%), vitamin C (99%), urea (ACS ≥ 99%), sodium hydroxide (NaOH, ACS ≥ 98%), acetone, methanol, ethanol, chloroform, and other reagents were of analytical grade and obtained from Sigma–Aldrich (Milwaukee, WI, USA). Toluene (99.8%) and tetrahydrofuran (THF, 99%) were supplied by Acros Organics B.V.B.A. (The Hauge, Belgium).

### 2.2. Preparation of Cellulose/rGO Composite Aerogels

**Figure 1:**
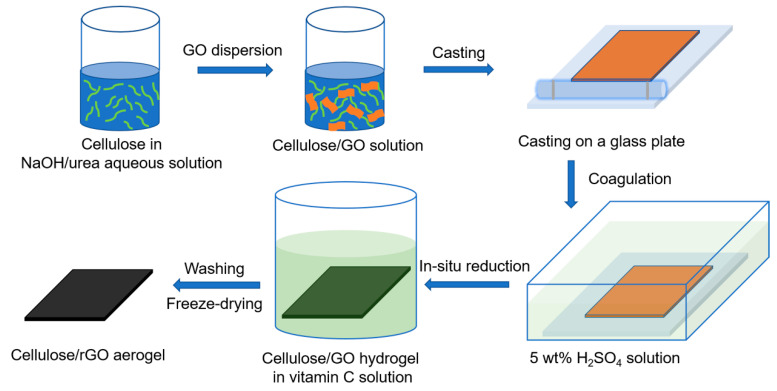
Schematic representation of the preparation of cellulose/rGO composite aerogels.

Figure 1 shows a schematic representation of the preparation of cellulose/rGO composite aerogels. A solution (100 g) of NaOH/urea/H_2_O (7: 12: 81 by weight) was prepared and pre-cooled to −12 °C. The designated amount of cellulose (4 g) was dissolved into the solution under mechanical stirring for 5 min. Subsequently, the calculated amount of GO dispersion (4 mg/mL) was added and the suspension was mixed for 5 min. using a mechanical stirrer. After degasification, the mixture was poured and cast on a glass plate by a glass tube. The thickness was controlled by two hoops at the ends of the glass tube. The mixed gel (about 1 mm in thickness) deposited on the glass plate was then soaked into a coagulation bath (800 mL) of 5 wt% H_2_SO_4_ at ambient temperature for 5 min. to coagulate and regenerate. The cellulose/GO hydrogels (about 0.5 mm in thickness; a certain degree of shrinkage occurred during the coagulation and regeneration process) were removed from the glass plate and then washed with de-ionized water for three days to clean off NaOH and urea. For the GO reduction, the cellulose/GO hydrogels were put in water solution (500 mL) of vitamin C (30 g/L) at 95 °C for 2 h. The resultant hydrogels were washed with de-ionized water for three days to clean off vitamin C. The cellulose/rGO hydrogels were rapidly frozen in liquid nitrogen for 5 min. and then lyophilised in a freeze drier (Alpha 1–2 LDplus, Martin Christ Gefriertrocknungsanlagen GmbH, Osterode, Germany) for 24 h to obtain the cellulose/rGO composite aerogels (about 0.5 mm thickness). By variation of the GO content related to the cellulose matrix to be 3, 5, or 8 wt%, we fabricated cellulose/rGO composite aerogels with various rGO loadings of 3, 5, and 8 wt%. Because the weight loss of GO during its reduction is unknown, we ignore it in the designation of the cellulose/rGO composites.

### 2.3. Characterization

The structure of the cellulose matrix was observed by characterizing cross-sections of the aerogel using an Ultra 55 scanning electron microscope (SEM, Carl Zeiss SMT AG, Oberkochen, Germany). The aerogels were stored in liquid nitrogen for 5 min. before cryo-fracturing, and the fractured surfaces were sputter-coated with a thin gold layer to hinder electrostatic charging.

### 2.4. Vapour Sensing Tests

**Figure 2:**
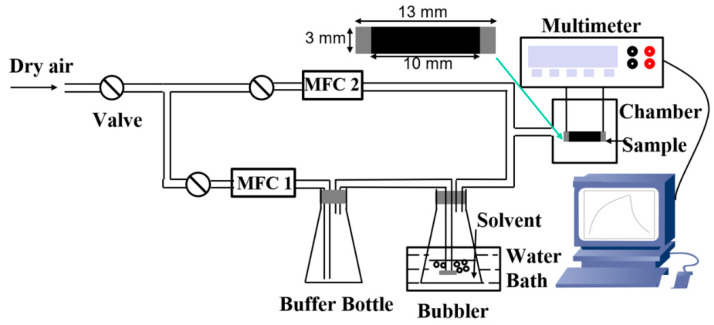
Schematic presentation of the experimental setup used for vapour sensing measurements.

The vapour sensing properties of the aerogels were evaluated by recording their electrical responses when exposed to alternating flows of dry air and vapour. The samples used as vapour sensors (13 mm × 3 mm × 0.5 mm) were cut from cellulose/rGO composite aerogels and coated with highly conductive silver paste (Acheson Electrodag 1415, Electron Microscopy Science, Hatfield, PA, USA) at the sample ends to obtain sufficient contact between electrodes and aerogels. The effective dimension of the part exposed to vapours was 10 mm × 3 mm × 0.5 mm. The measurements were carried out in a homemade measuring system (Figure 2). A controlled concentration of vapours was delivered to the chamber where the aerogel was placed for its analysis. Dry air functions as carrier and diluent for the vapours. The vapour concentrations were controlled by the mass flow controllers MFC1 and MFC2. Equation (1) calculated the actual vapour concentration.

(1) $$C\left(\%\right)=\frac{P_{i}}{P}\times \frac{f}{\left(f+F\right)}\times 100$$

*P* is the input air pressure (that is about ambient pressure), *P_i_* is the saturated partial pressure of the solvents at 25 °C (Table 1), and *f* and *F* are the gas flow rates of the saturated vapour and of dry air, respectively. The combined flow rate (*f* + *F*) was set to 30 L/h. The calculated saturated vapour concentrations of different vapours are incorporated in Table 1 in the result part. The resistance changes of the aerogels were recorded using a Keithley 2001 multimeter, which monitored the DC electrical resistance. To compare the performance of vapour sensors independently of the initial resistance, the relative electrical resistance change, *R_rel_*, is calculated according to Equation (2).

(2) $$R_{rel}\left(\%\right)=\frac{R_{t}-R_{0}}{R_{0}}\times 100$$

*R_t_* represents the transient resistance at time *t* when exposed to vapours or dry air and *R_0_* the initial resistance of the aerogel before starting the first exposure to the vapour after rinsing the sample for 200 s with dry air. The sensing tests were performed for water and organic vapours (methanol, ethanol, acetone, chloroform, tetrahydrofuran (THF), and toluene), all at 25 °C. Each vapour/dry air cycle consists of an exposure interval of 400 s, followed by a recovery interval of 200 s in dry air.

## 3. Results and Discussion

**Figure 3:**
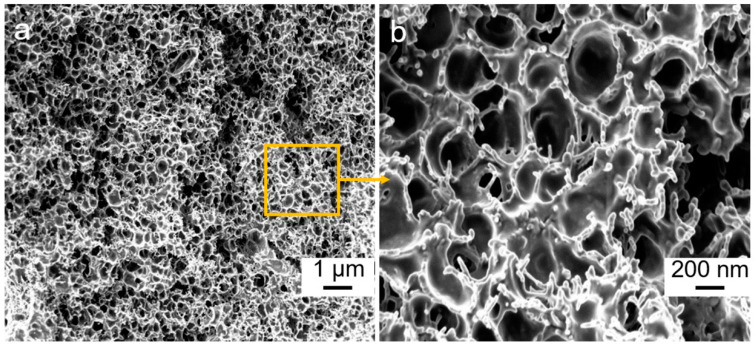
Scanning electron microscope (SEM) (**a**,**b**) of the cross-section of cellulose/rGO (5 wt%) composite aerogels.

Figure 3 shows the microscopic structure of cellulose/rGO (5 wt%) composite aerogels. The thickness of the composite aerogels is about 0.5 mm, being nearly the same as that of the hydrogel samples. The SEM images demonstrate that all of the aerogels possess open and highly porous networks with pore diameters of 200–500 nm.

In our previous work, Raman studies on the corresponding cellulose/GO and cellulose/rGO composites showed that the intensity ratio of the D-band to the G-band, which is commonly used to evaluate the reduction of GO, is about 1.52 for rGO composites and higher than about 1.06 for GO composites [37]. Such ratios can also be assumed for the composite aerogels, as only the drying method (room temperature drying for the composites versus freeze-drying for the aerogels) is changed. Although it is generally expected that during in situ chemical reduction, the D/G intensity ratio should decrease as the number of sp^3^ defects decreases due to the reduction, the opposite effect is often found in the literature. This has been explained by the concomitant reduction in the size dimensions of the sp^2^ domains in the plane [38,39], resulting in a large number of edges that are the reason for increased D-band intensities. Vitamin C cannot reduce GO completely, but sufficiently to obtain conductive rGO suitable for piezoresistive sensing applications.

**Figure 4:**
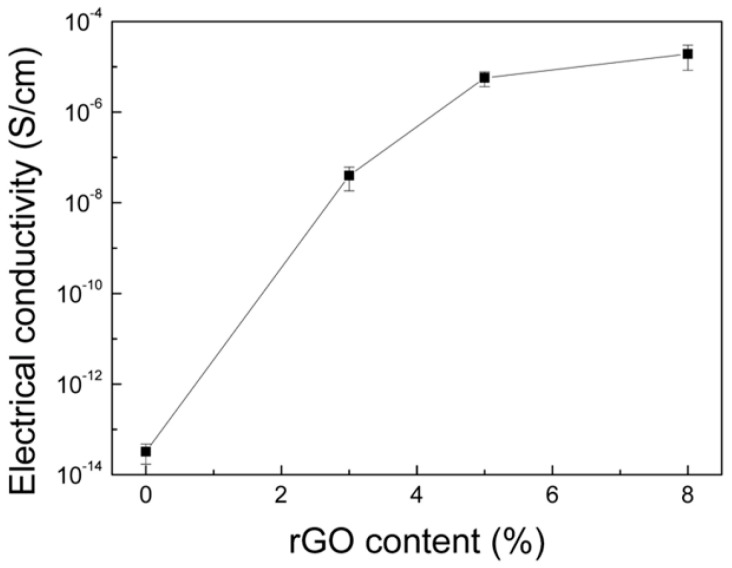
Electrical conductivity of cellulose/rGO aerogels in dependence on rGO content.

The electrical conductivity of cellulose/rGO composite aerogels can be adjusted by changing the content of rGO. The lowest amount of added rGO (3 wt%) is already above the percolation concentration and causes conductivity, as shown in Figure 4. The electrical conductivity is significantly enhanced with the increasing rGO content and reaches 1.9 × 10^−5^ S cm^−1^ in the case of the cellulose/rGO (8 wt%) aerogel. The electrical conductivity of cellulose/rGO confirms the success of the chemical reduction of GO to rGO and enables its application in vapour sensing.

**Figure 5:**
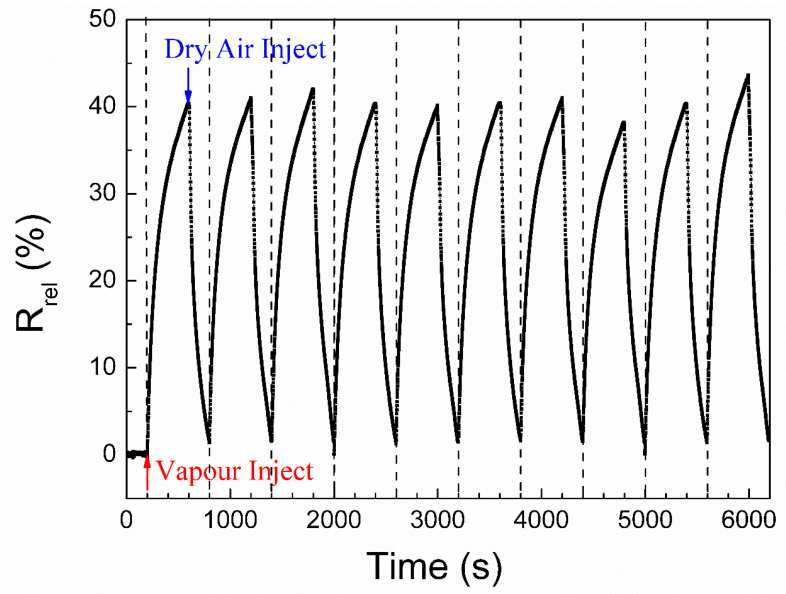
The electrical resistance change of cellulose/rGO (8 wt%) aerogels to saturated methanol vapour (C_i_ = 27.6%) at 25 °C.

The response and recovery of the electrical signal are significant factors for the applicability of electrically conductive materials as vapour sensors. Figure 5 displays the resistance response of cellulose/rGO (8 wt%) aerogels to saturated methanol vapour. Before the exposure to methanol vapour, the resistance change was recorded for 200 s under dry air to obtain an unwavering *R_0_*. Then, the aerogel was exposed to methanol vapour and dry air for ten cycles. As we can see, once the aerogel is contacted with methanol vapour, the *R_rel_* value of the aerogel immediately increases and reaches approximately 20% after about 50 s. After 400 s the *R_rel_* value reaches about 40%. As shown in Figure S1 (Supplementary Materials), *R_rel_* reached a plateau value (about 45%) after about 1000 s of exposure. Notedly, it needs longer time than the applied 400 s to reach the plateau for methanol vapour.

Very consistent results were observed in each cycle, as can be seen from Figure 5. The response levels of the sensor and maximum resistance change (about 40%) are quite stable and reproducible after repeated exposure to methanol vapour, and the recovery after the drying in air is always nearly complete, demonstrating the high repeatability of the cellulose/rGO aerogel in methanol sensing. Clearly, the cellulose/rGO aerogels exhibit a positive vapour coefficient to methanol vapour, which is also found in other rGO-based materials for vapour or gas sensing [11,40,41]. It is proposed that the electron conduction within this vapour sensor is due to the electron transfer between the rGO sheets. The open-porous structure of the cellulose matrix provides some advantages for vapour diffusion and penetration because of their large surface area and numerous vapour channels. Therefore, the swelling and de-swelling of the cellulose matrix as well as adsorption and desorption of the vapour molecules on the rGO sheets are fast and effective, which is favourable for triggering the electrical resistance changes.

**Figure 6:**
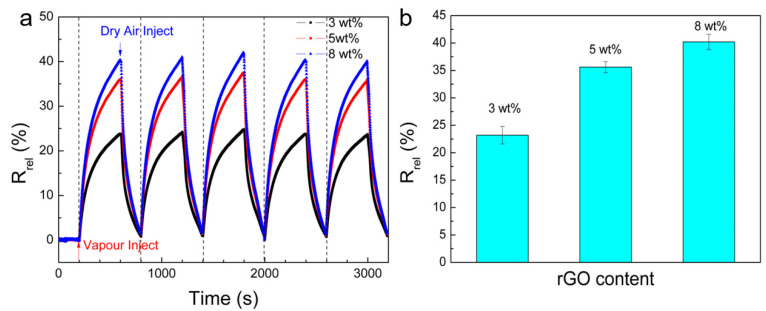
(**a**) *R_rel_* of cellulose/rGO aerogels with different rGO contents of 3 wt%, 5 wt% and 8 wt% during cyclic exposure to saturated methanol vapour (C_i_ = 27.6%) at 25 °C; and (**b**) the mean of the maximum *R_rel_* of five cyclic exposures to methanol vapour for 400 s.

Figure 6 shows the electrical responses of the cellulose/rGO aerogels containing 3 to 8 wt% rGO to methanol vapour. The mean of the maximum *R_rel_* of five cycles is 23% for the composite with the lowest rGO content of 3 wt% and increases to 35.3% and 40.2% for the composites when the rGO content is raised to 5 wt% and 8 wt%, respectively (Figure 6). Such behaviour is not expectable for the mostly observed mechanism of conductive network destruction due to network swelling, which commonly dominates the vapour and solvent sensing mechanism of CPCs. Typically, at higher filler loadings the network becomes denser and larger swelling is needed in order to destroy the conductive networks. Therefore, the strongest electrical response is expected at filler concentrations just above the electrical percolation concentration. However, a similar increase in electrical response with increased filler contents were reported for CPC based on polystyrene and multi-walled carbon nanotubes (MWCNT) [42] or polyurethane and carbon black (CB) [43]. Both of the systems show higher maximum electrical response when the content of conductive fillers increases and only at electrical filler concentrations far above the electrical percolation concentration the swelling mechanism becomes dominating. The unusual behaviour is explained by changes in the conduction mechanism [39,40] with raising filler contents and with specific solvent interactions to matrix components with different polarities [39].

**Figure 7:**
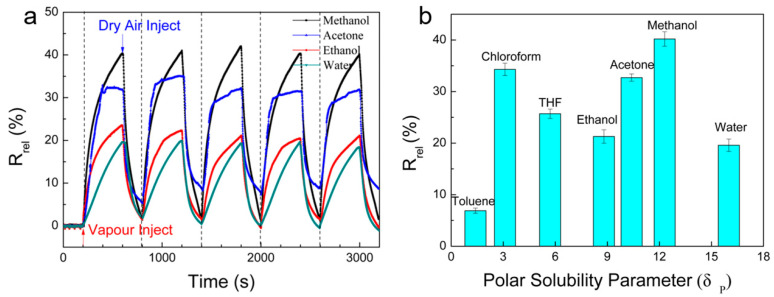
(**a**) *R_rel_* of cellulose/rGO (8 wt%) aerogels during five saturated vapour/air exposure cycles at 25 °C (saturated methanol vapour (C_i_ = 27.6%); saturated water vapour (C_i_ = 3.2%); saturated ethanol vapour (C_i_ = 7.9%); saturated acetone vapour (C_i_ = 30.2%)); and, (**b**) the mean of the maximum *R_rel_* of five exposures to vapours for 400 s versus the polar solubility parameter.

Because the ability to differentiate between different vapours is an important factor for the evaluation of the vapour sensor performance, seven different solvent vapours, namely methanol, ethanol, acetone, water, chloroform, toluene, and tetrahydrofuran (THF), have been used as analyte (Table 1). Figure 7a shows the responses of cellulose/rGO (8 wt%) aerogels to four different vapours during five exposure cycles. All of the curves show a positive vapour coefficient and good reproducibility. Moreover, the difference in the maximum resistance changes and the curve shape demonstrate different interactions between the aerogel and vapour molecules. Therefore, the sensing tests allow distinguishing different vapours. For example, once the aerogel is in contact with acetone vapour, *R_rel_* increased immediately and *R_rel_* reached a plateau value. *R_rel_* can hardly increase further since the rGO surface sites are already occupied by the acetone molecules reaching a saturated state. Swelling that will continue during exposure only has a negligible effect on conductivity and the increase in resistivity is dominated by the vapour adsorption. Otherwise, *R_rel_* increases steadily during exposure to water molecules. Cellulose contains plenty hydroxyl groups (Figure S2 (Supplementary Materials)), which may form hydrogen bonds with water. However, these bonds are not easily accessible to water since the cellulose hydroxyl groups form strong intramolecular bonds. Thus, the water absorption is slow, resulting in slow but continuous swelling of the cellulose matrix.

Similar ability to differentiate between different vapours by the shape of the resistance response curve was also found in cellulose-carbon nanotube (CNT) aerogel systems [36]. These composites exhibited similar sensitivity and even faster response than our studied system. Possibly, in the CNT containing system, the absorption/desorption of vapour molecules on the CNT is more dominating over the swelling/deswelling mechanism of the resistance change. The larger anisotropy of the CNT has a stronger stabilizing effect of the CPC than the rGO, making the system less accessible to polymer swelling. The balance between swelling and adsorption on the conductive filler surface is especially important in systems where a swellable matrix is coated with a conductive filler network like in TPU filaments with an anchored CNT network at the surface. This morphology resulted in in relative resistance changes from few 10%, as in our aerogels up to few 1000%, depending on the CNT network density and the swellability of the thin TPU filaments by the different vapours [44]. The high surface to volume ratio of fibres can cause fast response, even if the conductive filler is completely covered by the matrix polymer, as shown for poly(styrene-co-butadiene-co-styrene) (SBS) fibres with rough surface filled with different amounts of few layer graphene (FLG) [45] or CNT [46]. Additionally, here, some 100% resistance changes with fast response and good recovery were observed. Even more, since the SBS matrix consists of segments of polystyrene and polybutadiene, exhibiting different solubility parameters, these SBS/FLG fibres and SBS/CNT fibres can be used as sensors for polar and non-polar vapours. The resistance response is less when CNTs are used as conductive filler since the CNTs restrict more the matrix swelling and CNT/CNT contacts are less easy to disconnect than contacts between less anisotropic fillers, like FLG.

In contrast to the aerogels and fibres, compact sensor films that are based on CPCs with carbon fillers have much longer response times to vapour and exhibit poor recovery due to the longer penetration paths of the vapour molecules when swelling and deswelling the CPC [47,48]. This was found for thermoplastic polycarbonate-based composites [47] or in rubber-based systems, such as styrene butadiene rubber [48] with CNTs and carbon black as well as their mixtures.

**Table 1:** The mean of the maximum relative electrical resistance change (*R_rel_*) after 400 s at 25 °C and characteristics of cellulose [49] and solvents [50]: saturated partial pressure (*P_i_*), calculated saturated vapour concentration (C_i_), molar volume (V_mol_), Hansen solubility parameters for dispersion (δ_D_), polarity (δ_P_), hydrogen bonding (δ_H_), and calculated solubility parameter “distance” (D_SP_).

Polymer/ Solvent	R_rel_ (%)	P_i_ (kPa)	C_i_ (%)	V_mol_ (cm^3^/mol)	δ_D_ (MPa^−0.5^)	δ_P_ (MPa^−0.5^)	δ_H_ (MPa^−0.5^)	D_SP_ (MPa^−0.5^)
Cellulose					24.4	14.9	30.9	
Water	19.6	3.2	3.2	18.0	15.5	16.0	42.3	21.2
Methanol	40.2	28.0	27.6	40.7	15.1	12.3	22.3	20.7
Ethanol	21.3	8.0	7.9	58.5	15.8	8.8	19.4	21.6
Acetone	32.7	30.6	30.2	77.0	15.5	10.4	7.0	30.1
Toluene	6.9	3.8	3.8	106.9	18.0	1.4	2.0	34.4
THF	25.7	23.5	23.2	81.7	16.8	5.7	8.0	29.0
Chloroform	34.3	80.7	79.6	80.7	17.3	3.1	5.7	31.2

Hansen solubility parameters (Table 1) are a useful tool to estimate the miscibility of polymers and solvents that are caused by dispersion (Van der Waals) forces (δ_D_), polar forces (δ_P_), and hydrogen bonds (δ_H_) [50]. The idea behind is that “like dissolves like” and, thus, as smaller their solubility parameter “distance” D_SP_, calculated from their respective partial solubility parameters (Equation (3)), as better is the solubility (or swellability) of the polymer in the corresponding solvent [50].

(3) $$D_{SP}={\left[4{\left(\delta_{D}-\delta_{D}\right)}^{2}+{\left(\delta_{P}-\delta_{P}\right)}^{2}+{\left(\delta_{H}-\delta_{H}\right)}^{2}\right]}^{0.5}$$

Cellulose, as a polar material containing many hydroxyl groups, has affinity to polar solvents (such as water, ethanol, acetone, and methanol), but the correlation to the polarity of the solvent vapours is poor. Water with the highest polarity and highest hydrogen-binding energy has the lowest effect on the relative resistance change when compared to all other polar or hydrogen-bond forming solvent vapours. The reason is that also other factors influence the sensing behaviour like the vapour concentration and the molar volume of the vapour molecules. Water has the lowest saturated vapour concentration of only 3.2% (Table 1) and, related to this value, the *R_rel_* of 19.6% is relatively high. The low vapour pressure of water also explains the steady increase of *R_rel_* during the exposure to water molecules (Figure 7a), where no equilibrium is reached after 400 s. Despite the strong water-cellulose interactions the recovery is fast and nearly complete after rinsing for 200 s with dry air. The low molar volume of water is favourable for the water diffusion in the polymer and therefore for the swelling and deswelling. Otherwise, vapours with lower polarity and low ability to hydrogen bonds, such as chloroform and THF, show similar relative electrical resistance change response like polar vapours, with the *R_rel_* value being about 25.7 and 34.3%. These solvents have high vapour pressure, resulting in fast filling of the aerogel pores, where they can interact with the matrix and rGO. Consequentially, the vapour with the lowest partial pressure, highest molar volume, and lowest solubility parameter, in this study toluene, has the smallest effect on the resistivity of the aerogel.

**Figure 8:**
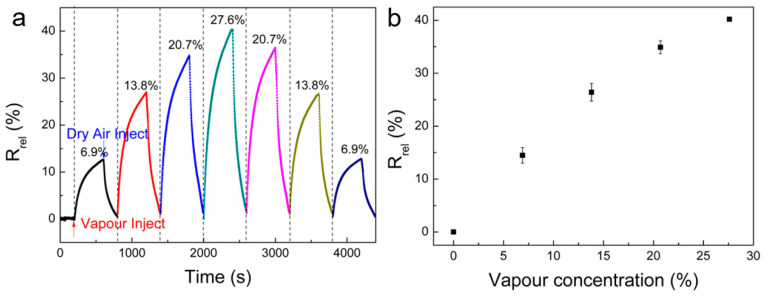
(**a**) *R_rel_* of the cellulose/rGO (8 wt%) aerogel upon sequential exposure to various methanol vapour concentrations at 25 °C; and, (**b**) the relationship between methanol vapour concentration and *R_rel_* after 400 s of exposure.

Finally, the quantitative performance of cellulose/rGO (8 wt%) aerogels for vapour sensing was tested. Figure 8 displays the real-time relative resistance measurement of the aerogel exposed to various methanol vapour concentrations at room temperature. The cycling test is carried out with different methanol vapour concentrations in order of 6.9%, 13.8%, 20.7%, and 27.6%, and then conversely from high to low concentration. Each vapour/dry air cycle is performed by an exposure interval of 400 s followed by a recovery interval of 200 s in dry air. The aerogel shows good recovery and fast response for all methanol vapour concentrations. A clear correlation of the *R_rel_* is observed with increasing and, more important, with decreasing methanol vapour concentration. This demonstrates that the aerogel has the potential for quantitative tests of vapour contents at room temperature. Moreover, the *R_rel_* values show nearly linear growth with methanol vapour concentrations, as shown in Figure 8b, while an exponential increase is observed in other materials as vapour sensors, where the swelling dominates the relative resistance change [51,52]. For such CPC sensor types, the exponential increase could be explained by a well-established sorption model that was reported by Feller et al. [53] and is due to solubility increase along with cellulose swelling and penetrant clustering of vapour molecules [44]. Here, this model is not applicable, since adsorption and desorption on the conductive filler dominate the resistance change.

## 4. Conclusions

This contribution demonstrates, for the first time, the possibility to exploit cellulose/rGO composite aerogels as vapour sensors. These composite aerogels exhibit fast response and good recovery, are highly sensitive, and exhibit well reproducibility. It was shown that discrimination of vapours is possible by analysing the shape and height of the resistance response of the aerogel when exposed to different vapours. A nearly linear response could be obtained in a wide range of methanol vapour concentration. Thus, cellulose/rGO composite aerogels are suitable for vapour quantification. The inexpensive, easy, green, and scalable preparation of this new type of vapour sensors is expected to pave a new avenue for vapour sensing applications. By combining the highly open-porous cellulose matrix with the nature of rGO, cellulose/rGO composite aerogels may also serve as a new platform for designing a new class of multifunctional sensing materials.

## Supplementary Materials

The following are available online at https://www.mdpi.com/2079-4991/10/9/1729/s1, Figure S1: The electrical resistance change of cellulose/rGO (8 wt%) aerogel during exposure to saturated methanol vapour (C_i_ = 27.6%) at 25 °C and its recovery in dry air, Figure S2: The chemical structure of cellulose.
