# Correction: Chen et al. Aerogels Based on Reduced Graphene Oxide/Cellulose Composites: Preparation and Vapour Sensing Abilities. *Nanomaterials* 2020, *10*, 1729

**DOI:** 10.3390/nano15010024

**Published:** 2024-12-27

**Authors:** Yian Chen, Petra Pötschke, Jürgen Pionteck, Brigitte Voit, Haisong Qi

**Affiliations:** 1 Leibniz-Institut für Polymerforschung Dresden e. V. (IPF), 01069 Dresden, Germany; chen@ipfdd.de (Y.C.); pionteck@ipfdd.de (J.P.); voit@ipfdd.de (B.V.); 2 Organic Chemistry of Polymers, Faculty of Chemistry and Food Chemistry, Technische Universität Dresden, 01062 Dresden, Germany; 3 State Key Laboratory of Pulp and Paper Engineering, South China University of Technology, Guangzhou 510640, China; 4 Guangdong Engineering Research Center for Green Fine Chemicals, South China University of Technology, Guangzhou 510640, China

## Error in Figure

In the original publication [1], there was a mistake in Figure 4 as published. The Raman spectra data contained in Figure 4a were found to be accidently duplicated from another paper, where they were used for a different processing state of the composites (Chen, Y.; Pötschke, P.; Pionteck, J.; Voit, B.; Qi, H. Smart cellulose/graphene composites fabricated by *in situ* chemical reduction of graphene oxide for multiple sensing applications. *J. Mater. Chem. A* **2018**, *6*, 7777–7785. https://doi.org/10.1039/C8TA00618K). Therefore, Figure 4a is removed. The corrected Figure 4 appears below.

## Text Correction

There was an error in the original publication. Following the removal of Figure 4a, some parts of the text have been removed or revised. A correction has been made to 2. Experimental, 2.3. Characterization, Paragraph 1:

“The structure of the cellulose matrix was observed by characterizing cross-sections of the aerogel using an Ultra 55 scanning electron microscope (SEM, Carl Zeiss SMT AG, Oberkochen, Germany). The aerogels were stored in liquid nitrogen for 5 min. before cryo-fracturing, and the fractured surfaces were sputter-coated with a thin gold layer to hinder electrostatic charging.”

A correction has been made to 3. Results and Discussion, Paragraph 2:

*“*In our previous work, Raman studies on the corresponding cellulose/GO and cellulose/rGO composites showed that the intensity ratio of the D-band to the G-band, which is commonly used to evaluate the reduction of GO, is about 1.52 for rGO composites and higher than about 1.06 for GO composites [37]. Such ratios can also be assumed for the composite aerogels, as only the drying method (room temperature drying for the composites versus freeze-drying for the aerogels) is changed. Although it is generally expected that during in situ chemical reduction, the D/G intensity ratio should decrease as the number of sp^3^ defects decreases due to the reduction, the opposite effect is often found in the literature. This has been explained by the concomitant reduction in the size dimensions of the sp^2^ domains in the plane [38,39], resulting in a large number of edges that are the reason for increased D-band intensities. Vitamin C cannot reduce GO completely, but sufficiently to obtain conductive rGO suitable for piezoresistive sensing applications.”

A correction has been made to 3. Results and Discussion, Paragraph 3:

“The electrical conductivity of cellulose/rGO composite aerogels can be adjusted by changing the content of rGO. The lowest amount of added rGO (3 wt%) is already above the percolation concentration and causes conductivity, as shown in Figure 4. The electrical conductivity is significantly enhanced with the increasing rGO content and reaches 1.9 × 10^−5^ S cm^−1^ in the case of the cellulose/rGO (8 wt%) aerogel. The electrical conductivity of cellulose/rGO confirms the success of the chemical reduction of GO to rGO and enables its application in vapour sensing.”

The authors state that the scientific conclusions are unaffected. This correction was approved by the Academic Editor. The original publication has also been updated.

## Figures and Tables

**Figure 4:**
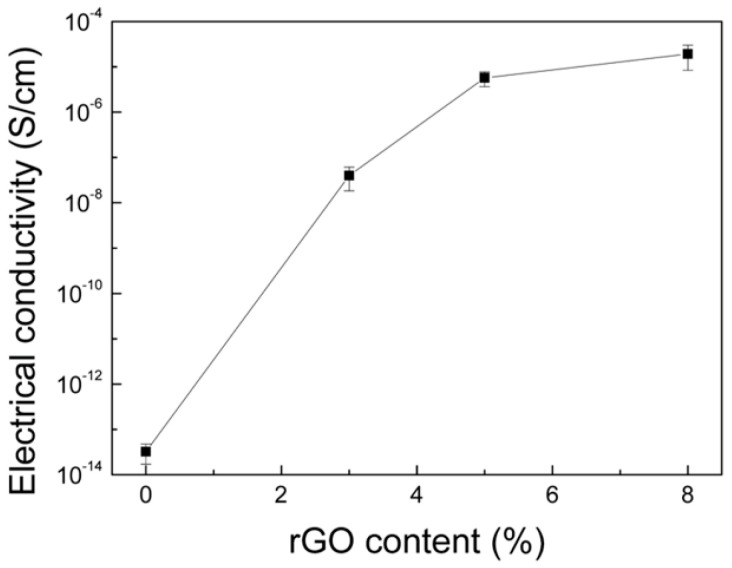
Electrical conductivity of cellulose/rGO aerogels in dependence on rGO content.
