# Supplementary material for: Aerogels Based on Reduced Graphene Oxide/Cellulose Composites: Preparation and Vapour Sensing Abilities
Source: Nanomaterials (Basel). 2020 Aug 31;10(9):1729. doi: 10.3390/nano10091729 (PMC7560231; doi:10.3390/nano10091729)
Supplement: Supplementary file 1 [file nanomaterials-10-01729-s001.pdf]

Supplementary Materials

Aerogels Based on Reduced Graphene Oxide/Cellulose Composites: Preparation and Vapour Sensing Abilities

Yian Chen [1,2], Petra Pötschke [1,*], Jürgen Pionteck [1], Brigitte Voit [1,2] and Haisong Qi [3,4,*]

[1] Leibniz-Institut für Polymerforschung Dresden e. V. (IPF), 01069 Dresden, Germany; chen@ipfdd.de (Y.C.); pionteck@ipfdd.de (J.P.); voit@ipfdd.de (B.V.)

[2] Organic Chemistry of Polymers, Faculty of Chemistry and Food Chemistry, Technische Universität Dresden, Organic Chemistry of Polymers, 01062 Dresden, Germany

[3] State Key Laboratory of Pulp and Paper Engineering, South China University of Technology, Guangzhou 510640, China

[4] Guangdong Engineering Research Center for Green Fine Chemicals, South China University of Technology, Guangzhou 510640, China

* Correspondence: poe@ipfdd.de (P.P.); qihs@scut.edu.cn (H.Q.)

Resistance Response of Cellulose/rGO (8 wt%) Aerogel to Saturated Methanol Vapour at Room Temperature During Long Term Exposure

As shown in Figure S1, the resistance response of cellulose/rGO (8 wt%) aerogels to saturated methanol vapour at room temperature can reach a plateau after about 1000 s of exposure. The recovery from this state is rather fast and is almost complete after 200 s. For better comparison with other vapours and evaluation of vapour sensitivity in shorter times, each vapour/dry air cycle is performed by an exposure interval of 400 s followed by a recovery interval of 200 s in dry air.

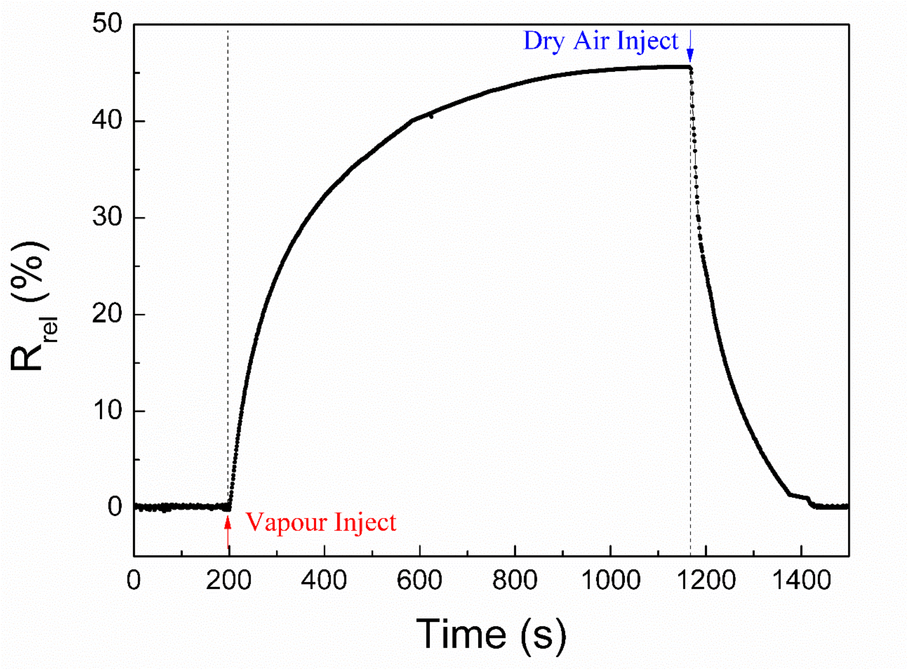

Figure S1. The electrical resistance change of cellulose/rGO (8 wt%) aerogel during exposure to saturated methanol vapour (C_i = 27.6%) at 25 °C and its recovery in dry air.

Structure of Cellulose

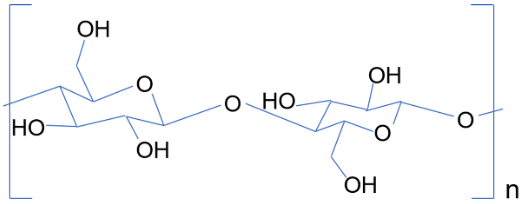

Figure S2. The chemical structure of cellulose.

Increased interaction between H-bond forming solvents and cellulose are expected due to the presence of OH-groups in each monomer unit, even if they are not easy accessible because of strong intramolecular hydrogen bridges.
